# Utilization of CT scanning associated with complex spine surgery

**DOI:** 10.1186/s12891-017-1420-9

**Published:** 2017-01-31

**Authors:** Vikas V. Patel, Gunnar B. J. Andersson, Steven R. Garfin, Donald L. Resnick, Jon E. Block

**Affiliations:** 1 The Spine Center, University of Colorado Hospital, Denver, CO USA; 2 Midwest Orthopaedics, RUSH University Medical Center, Chicago, IL USA; 3 Department of Orthopaedic Surgery, University of California, San Diego, CA USA; 4 Department of Radiology, University of California, San Diego, CA USA; 5 2210 Jackson Street, Ste. 401, San Francisco, CA 94115 USA

**Keywords:** CT scanning, Spine surgery, Fusion, Instrumentation, Discectomy, Laminectomy, Cancer

## Abstract

**Background:**

Due to the risk associated with exposure to ionizing radiation, there is an urgent need to identify areas of CT scanning overutilization. While increased use of diagnostic spinal imaging has been documented, no previous research has estimated the magnitude of follow-up imaging used to evaluate the postoperative spine.

**Methods:**

This retrospective cohort study quantifies the association between spinal surgery and CT utilization. An insurance database (Humana, Inc.) with ≈ 19 million enrollees was employed, representing 8 consecutive years (2007–2014). Surgical and imaging procedures were captured by anatomic-specific CPT codes. Complex surgeries included all cervical, thoracic and lumbar instrumented spine fusions. Simple surgeries included discectomy and laminectomy. Imaging was restricted to CT and MRI. Postoperative imaging frequency extended to 5-years post-surgery.

**Results:**

There were 140,660 complex spinal procedures and 39,943 discectomies and 49,889 laminectomies. MRI was the predominate preoperative imaging modality for all surgical procedures (median: 80%; range: 73–82%). Postoperatively, CT prevalence following complex procedures increased more than two-fold from 6 months (18%) to 5 years (≥40%), and patients having a postoperative CT averaged two scans. For simple procedures, the prevalence of postoperative CT scanning never exceeded 30%.

**Conclusions:**

CT scanning is used frequently for follow-up imaging evaluation following complex spine surgery. There is emerging evidence of an increased cancer risk due to ionizing radiation exposure with CT. In the setting of complex spine surgery, actions to mitigate this risk should be considered and include reducing nonessential scans, using the lowest possible radiation dose protocols, exerting greater selectivity in monitoring the developing fusion construct, and adopting non-ferromagnetic implant biomaterials that facilitate MRI postoperatively.

**Electronic supplementary material:**

The online version of this article (doi:10.1186/s12891-017-1420-9) contains supplementary material, which is available to authorized users.

## Background

The advent and continuous refinement of advanced imaging techniques, specifically computed tomography (CT) and magnetic resonance imaging (MRI), have revolutionized the diagnosis and surgical management of spinal disorders. These imaging techniques provide exquisite detail and characterization of anatomical structures and degenerative changes as well as postoperative evaluation of surgical interventions previously unachievable with standard radiography. Due, in part, to these technical advancements, the rates of advanced spinal imaging have increased markedly over the past several decades [1, 2]. However, it has been estimated that one-third of advanced spinal imaging may be inappropriate or unnecessary, and physician panels convened by the American Board of Internal Medicine Foundation have identified spinal imaging among the top 5 commonly overused procedures [1, 3]. In fact, adults with no back or radicular pain often show spinal abnormalities on advanced imaging and, indeed, the use of these imaging modalities to improve diagnosis has been implicated as a primary factor in the equally dramatic increase in complex spinal surgeries [3–7].

Recognizing the sizable increases in advanced imaging across all areas of medicine [8, 9], the American College of Radiology launched the Image Wisely campaign to curb unnecessary imaging and encourage the use of the minimum amount of radiation needed to perform the indicated test [10]. Unfortunately, the society’s appropriateness criteria have had limited impact on reducing the frequency of nonessential imaging due, in part, to the lack of specificity in identifying which imaging studies are appropriate based on the preponderance of clinical data [11].

Reducing the frequency of unnecessary spinal imaging, particularly techniques that expose patients to high levels of ionizing radiation such as CT, hinges on our ability to pinpoint areas of previously unidentified and/or unquantifiable imaging utilization. While much has been published about the rise in preoperative diagnostic spinal imaging, there has been scant mention of the magnitude of spinal imaging that is undertaken postoperatively. The current study was conducted to estimate how often and at what juncture advanced imaging, particularly CT scanning, is utilized to evaluate the postoperative spine.

## Methods

The objective of this retrospective cohort study was to examine and quantify the association between spinal surgery and the utilization of advanced imaging, particularly CT and MRI. In addition to determining the prevalence of preoperative diagnostic imaging, we specifically investigated the frequency and timing of postoperative follow-up imaging.

This study utilized an insurance-based administrative database of adjudicated patient claims records (PearlDiver Technologies, West Conshohocken, PA, USA). This electronic database contains procedural volumes and demographics for patients based on specific Current Procedural Terminology (CPT) codes. All data were de-identified and anonymous, and were thus exempt from ethics committee approval [12]. The longitudinal data presented within this report were drawn from actual rates evident within a single managed healthcare system (Humana, Inc., Louisville, KY) representing 8 consecutive years from 2007 through the fourth quarter of 2014. Enrollees within this healthcare system resided mostly in the South (60%) and Midwest (25%) portions of the US. Imaging results are based upon incidence rates for those patients with a full follow-up period available. These files contain patient information identified by CPT coding related to spinal procedures and imaging procedures. All data are Health Insurance Portability and Accountability Act (HIPAA) compliant to protect patient privacy.

Two groups of spine surgery patients were identified by specific CPT codes and segmented based on the complexity of the procedure. “Complex” surgical procedures included cervical fusion, lumbar fusion and other long-segment fusion procedures. The latter group consisted of fusion procedures spanning multiple segments indicated mostly for spinal deformities (e.g., scoliosis, Scheuermann’s disease, etc.). All complex procedures involved the concomitant implantation of metallic instrumentation including plates, rods, screws, and interbody devices, often in combination. No distinctions were made regarding surgical approach/technique, i.e., anterior, posterior, interbody, circumferential. “Simple” surgical procedures included discectomy and laminectomy decompression procedures at all levels without instrumentation. Disc arthroplasty procedures were not included as annual procedural counts were less than 200 patients. Specific CPT search codes for each surgical procedure are provided in Additional file 1.

Corresponding imaging events were restricted solely to CT and MRI, and captured using anatomic-specific CPT codes. Advanced imaging CPT search codes are also provided in Additional file 1. To determine preoperative imaging prevalence, an interval of 6 months prior to the index surgical procedure was chosen as this duration corresponds with the period routinely mandated by US federal regulators and medical societies as sufficient to exhaust conservative medical management. Postoperative imaging frequency extended to a period of 5 years after the index surgical procedure.

To adjust for the wide geographic variability in surgical practice patterns [13], estimates of US patient volumes for each surgical or imaging procedure were extrapolated using an algorithm based upon the prevalence of each procedure evident within the Humana patient record set and the Medicare Standard Analytical file for populations under sixty-five and sixty-five plus, respectively. Changes in procedural frequency between 2007 and 2014 were estimated using standard compound annual growth rate (CAGR) computations.

## Results

The Humana healthcare database comprised adjudicated claims for 18,620,198 unique patients for the study period of 2007–2014 with an annual median number of enrollees of 6,779,769 (range: 5,412,897–9,434,477). The number of patients (procedures) identified by specific CPT codes for the overall study duration was as follows: cervical fusion, 29,888 (31,121); lumbar fusion, 37,579 (40,199); other long-segment fusion procedures, 63,967 (69,340); discectomy, 38,887 (39,943); and, laminectomy, 49,022 (49,899). Patient demographics are provided in Table 1 for each surgical procedure separately. The largest percentage of surgical interventions occurred in patients > 65 years of age irrespective of operative procedure, with 70% of laminectomy patients included in this age group.

**Table 1:** Background Characteristics by Procedure

Variable	Cervical Fusion (*N* = 31,121)	Lumbar Fusion (*N* = 40,199)	Other (*N* = 69,340)	Discectomy (*N* = 39,943)	Laminectomy (*N* = 49,889)
Demographics					
Female Sex, n (%)	15,843 (51)	23,524 (59)	38,402 (55)	19,396 (49)	25,101 (50)
Age, n (%), yr					
< 20	0 (0)	125 (0)	595 (1)	109 (0)	181 (0)
20–34	0 (0)	616 (2)	1,166 (2)	2,069 (5)	680 (1)
35–49	5,550 (18)	3,569 (9)	8,671 (13)	7,372 (18)	3,076 (6)
50–64	11,333 (36)	10,996 (27)	21,312 (31)	11,340 (28)	11,145 (22)
65+	13,494 (43)	24,558 (61)	37,094 (53)	19,044 (48)	34,808 (70)

From 2007–2014, the CAGR within the Humana data set for complex surgical procedures was 9.72%, 12.28% and 10.84% for cervical fusion, lumbar fusion and other long-segment fusion procedures, respectively. In comparison, the corresponding CAGR for simple surgical procedures was -1.35% for discectomy and 11.30% for laminectomy.

**Fig. 1:**
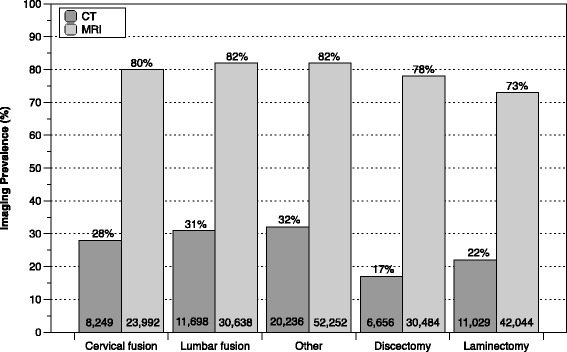
Preoperative imaging prevalence within 6 months of index surgery by procedure

Previous imaging prevalence within 6 months of the index surgical procedure is illustrated in Figure 1. MRI predominated as the primary preoperative advanced diagnostic imaging modality for all surgical procedures (median: 79%; range: 73–82%). Preoperative CT scanning prevalence was notably lower than MRI but, in general, utilization of diagnostic CT was qualitatively higher among patients undergoing complex surgical procedures (median: 30%; range: 28–32%) compared to simple surgical procedures (median: 20%; range: 17–22%).

**Fig. 2:**
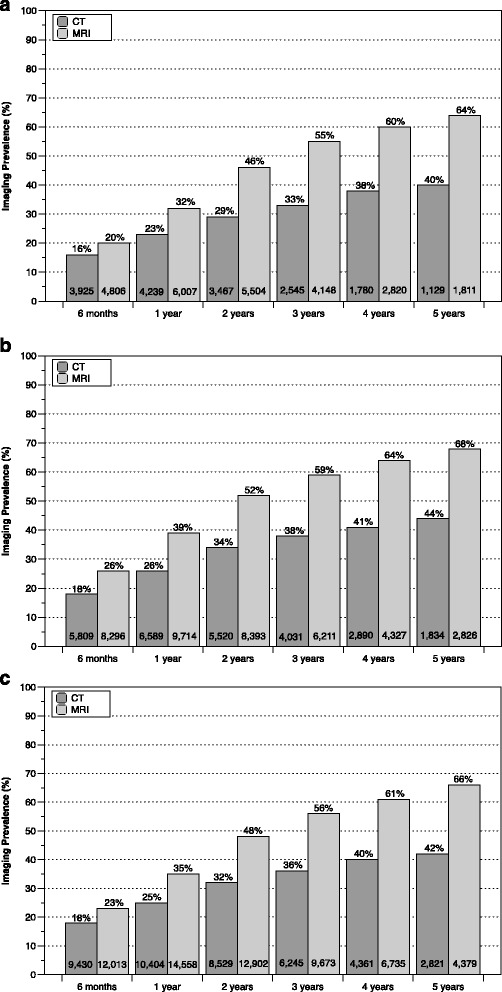
Postoperative imaging prevalence for complex surgical procedures by follow-up interval for cervical fusion (**a**), lumbar fusion (**b**) and other long-segment fusion procedures (**c**). The corresponding number of imaging events is provided at the bottom of each bar

**Fig. 3:**
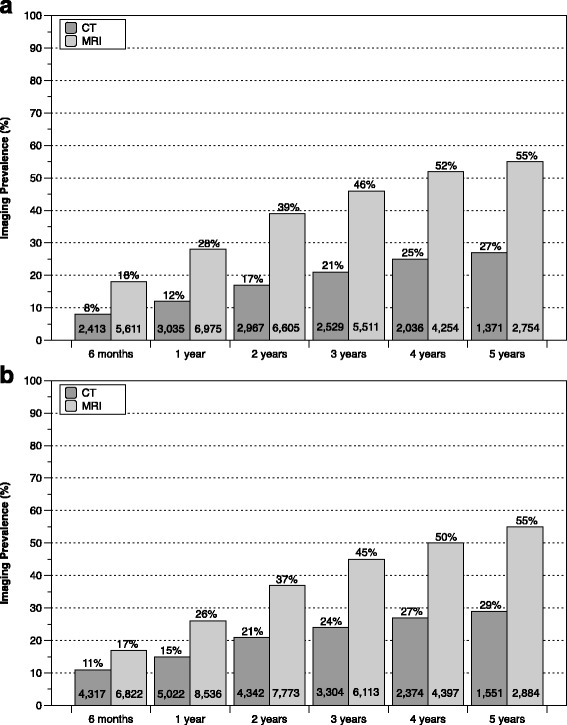
Postoperative imaging prevalence for simple surgical procedures by follow-up interval for discectomy (**a**) and laminectomy (**b**). The corresponding number of imaging events is provided at the bottom of each bar

Postoperative imaging prevalence through 5 years of follow-up for all surgical procedures is illustrated in Figures 2 and 3. For complex surgical procedures, CT scanning prevalence increased more than two-fold from 6 months to 5 years postoperatively, with approximately 30% of patients having at least one follow-up CT scan by 2 years and ≥ 40% of patients having a follow-up CT scan by 5 years (Figure 2). For simple surgical procedures, the prevalence of postoperative CT scanning was lower at all postoperative intervals compared to the prevalence associated with complex procedures (Figure 3). While there was an approximate three-fold increase in CT scanning prevalence over the 5-year follow-up period for simple surgical procedures, the frequency never exceeded 30% at any postoperative interval. For patients having postoperative CT scanning after a complex surgical procedure, the average number of CT imaging events after 5 years of follow-up was 1.97, 2.03 and 2.00 for cervical fusion, lumbar fusion and other long-segment fusion procedures, respectively. The corresponding average number of CT events was 1.68 for both discectomy and laminectomy over the same 5-year follow-up period.

For patients having a postoperative CT scan, Table 2 shows the distribution of CT scan frequency at 6 months postoperatively for each surgical procedure for each study year separately. There is a uniformly steady rise in the number of CT scans over the study observation period (2007 through 2014) with most patients receiving one CT scan by 6 months, but > 20% of patients received multiple CT scans at this early follow-up interval.

**Table 2:** CT Scan Frequency for each Procedure by Year: All Patients at 6 months Follow-up

Procedure	2007	2008	2009	2010	2011	2012	2013	2014
Cervical Fusion, n (%)	(*N* = 105)	(*N* = 135)	(*N* = 201)	(*N* = 209)	(*N* = 249)	(*N* = 291)	(*N* = 382)	(*N* = 339)
No. of Scans 1	81 (77)	107 (79)	142 (71)	156 (75)	174 (70)	214 (74)	297 (78)	274 (81)
2	17 (16)	18 (13)	40 (20)	39 (19)	47 (19)	53 (18)	64 (17)	48 (14)
3	* (*)	* (*)	11 (5)	* (*)	17 (7)	19 (7)	15 (4)	11 (3)
4+	* (*)	* (*)	* (*)	* (*)	11 (4)	* (*)	* (*)	* (*)
Lumbar Fusion, n (%)	(*N* = 119)	(*N* = 165)	(*N* = 220)	(*N* = 253)	(*N* = 335)	(*N* = 413)	(*N* = 521)	(*N* = 474)
No. of Scans 1	88 (74)	137 (83)	167 (76)	207 (82)	264 (79)	332 (80)	430 (83)	391 (82)
2	24 (20)	17 (10)	41 (19)	31 (12)	54 (16)	63 (15)	65 (12)	52 (11)
3	* (*)	* (*)	* (*)	* (*)	* (*)	11 (3)	22 (4)	22 (5)
4+	* (*)	* (*)	* (*)	* (*)	* (*)	* (*)	* (*)	* (*)
Other, n (%)	(*N* = 223)	(*N* = 305)	(*N* = 430)	(*N* = 461)	(*N* = 563)	(*N* = 690)	(*N* = 909)	(*N* = 798)
No. of Scans 1	169 (76)	246 (81)	317 (74)	358 (78)	420 (75)	528 (77)	722 (79)	646 (81)
2	39 (17)	37 (12)	84 (20)	70 (15)	96 (17)	117 (17)	134 (15)	99 (12)
3	* (*)	15 (5)	14 (3)	20 (4)	24 (4)	31 (4)	41 (5)	37 (5)
4+	* (*)	* (*)	15 (3)	13 (3)	23 (4)	14 (2)	12 (1)	16 (2)
Discectomy, n (%)	(*N* = 228)	(*N* = 314)	(*N* = 313)	(*N* = 392)	(*N* = 300)	(*N* = 264)	(*N* = 309)	(*N* = 279)
No. of Scans 1	154 (68)	243 (77)	233 (74)	281 (72)	224 (75)	196 (74)	242 (78)	214 (77)
2	56 (25)	55 (18)	55 (18)	78 (20)	53 (18)	51 (19	42 (14)	48 (17)
3	13 (6)	* (*)	14 (4)	21 (5)	11 (4)	* (*)	15 (5)	* (*)
4+	* (*)	* (*)	11 (4)	12 (3)	12 (4)	* (*)	* (*)	* (*)
Laminectomy, n (%)	(*N* = 249)	(*N* = 325)	(*N* = 419)	(*N* = 475)	(*N* = 577)	(*N* = 695)	(*N* = 809)	(*N* = 774)
No. of Scans 1	191 (77)	242 (74)	304 (73)	328 (69)	432 (75)	509 (73)	578 (71)	587 (76)
2	50 (20)	58 (18)	84 (20)	109 (23)	100 (17)	131 (19)	163 (20)	131 (17)
3	* (*)	13 (4)	17 (4)	27 (6)	35 (6)	32 (5)	38 (5)	29 (4)
4+	* (*)	12 (4)	14 (3)	11 (2)	* (*)	23 (3)	30 (4)	27 (3)

*Patient volumes fewer then 11 are denoted with an asterisk (*) to protect patient privacy

Extrapolating the Humana surgical prevalence rates to the entire US population for the most recent study year (2014) resulted in an estimated number of patients having complex procedures to be 292,950 for cervical fusion, 298,721 for lumbar fusion and 436,586 for other long-segment fusion procedures. In 2014, an estimated 211,529 and 267,513 patients had discectomies and laminectomies, respectively. Correspondingly, based on the advanced imaging CPT codes used in the current study, the estimated number of all spine-specific preoperative and postoperative CT scans and MRI studies in 2014 in the US for patients undergoing complex spine procedures was 9,180,589 and 8,880,972, respectively.

## Discussion

With approximately 80% prevalence, the current study documented that MRI was the predominant imaging modality to diagnose and characterize spinal disorders prior to surgery. Use of preoperative CT scanning occurred with much lower frequency, but was highest among patients scheduled for complex surgical procedures.

While there have been previous reports discussing the unique imaging requirements necessary to evaluate radiographically the postoperative spine [14, 15], this is the first study to our knowledge to quantify the magnitude and timing of CT scan utilization following simple and complex surgical interventions. The frequency of CT scanning postoperatively was noteworthy with approximately one-quarter of patients undergoing complex spinal procedures having a follow-up CT scan within one year of surgery, with the prevalence exceeding 40% by 5 years postoperatively. Importantly, patients having postoperative CT scans averaged approximately two such imaging events over a 5-year follow-up period, increasing the effects of ionizing radiation exposure. Postoperative CT scan utilization was lower among patients having simple surgical procedures, occurring at about one-half the frequency associated with complex surgical procedures during the initial postoperative year, but still approached almost 30% by 5 years of follow-up.

Why is the prevalence of postoperative CT scanning as high as it is and why is it greater in patients having complex surgical procedures? One reason may be that complex spine surgery invariably involves the implantation of bone graft materials coupled with metallic instrumentation to provide mechanical stability to support fusion as well as surgical insertion of spacers, cages, motion sparing and arthroplasty devices. The dramatically increased utilization of these devices has led to a new field of postoperative imaging assessment to evaluate these implants for breakage, loosening, migration, subsidence, and expulsion as well as to assess the status of the ossifying fusion mass and adjacent level disease [15]. Unfortunately, the presence of metallic instrumentation, often spanning multiple vertebral levels, may necessitate the use of CT scanning to evaluate postoperative neural compression as the image distortion with MRI may be too great [16–19]. This is particularly true for implants composed of stainless steel or cobalt-chrome [20]. Since simple surgical procedures such as discectomy and laminectomy do not involve the concomitant implantation of instrumentation, the CT scanning frequency is correspondingly lower.

With instrumented spinal fusion, CT is often the preferred postoperative evaluation method to elucidate putative sources of residual or worsening symptoms such as screw loosening/breakage and delayed healing/pseudoarthrosis of the developing fusion construct that cannot be visualized adequately with other modalities. However, screw loosening is an infrequent surgical complication and rarely associated with bothersome clinical symptoms [21]. Much of the early-term use of postoperative CT scanning is likely undertaken to confirm healing of the fusion mass. However, pseudoarthrosis is rare and almost all patients inevitably fuse with current interbody techniques, with a solid arthrodesis occurring in greater than 90% of patients [22, 23]. Moreover, fusion status has been shown to be a weak predictor of clinical outcomes with only a modest correlation to back symptoms [24].

Our concern about the marked utilization of postoperative CT scanning is amplified by the growing body of evidence that patients exposed to radiation in the range provided by a single CT scan have an increased cancer risk [25, 26]. Smith-Bindman et al [27] estimated that the lifetime attributable cancer risk from a single CT scan could be as high as 1 in 80 depending on dose and life expectancy. The radiation dose associated with a standard CT study of the spine ranges from 5–8 mSv which equates to approximately 400 to 550 chest X-rays [28].

In the Humana population, the estimated growth rate in complex surgical procedures was approximately 10% per year with almost one million spinal operations being undertaken nationwide when extrapolated to the entire US population. Applying our postoperative imaging prevalence rates to this estimate suggests that about 250,000 patients annually will be subjected to at least a single CT scan within one year of their surgery. Of the 80–100 million CT scans performed in the US annually [29], we estimated that approximately 9 million involve spinal imaging, much of it postoperative follow-up imaging. CT scanning rates overall are also growing at an annual rate of about 10% [29]. This combination of factors should underscore efforts to address this previously unidentified public health concern.

As documented in this study, a healthy percentage of patients having spine surgery are > 65 years of age and, due to their decreased life expectancy, have a lower attributable cancer risk from a single CT scan. However, complex spine surgery for disc degeneration, which affects a far younger cohort typically in their 40s, is growing at a dramatic rate [30]. In this population, the risk of cancer associated with postoperative CT scanning can be very real [31].

Our findings support the adoption of methods to curb nonessential CT scanning after spine surgery and make certain that the lowest possible dose is used to address the clinical question at hand [11, 25, 26]. To accomplish this, we advocate better coordination between surgeons and radiologic staff to ensure that scanning protocols are as parsimonious as possible. We also urge greater selectivity and judiciousness in the use of CT scanning to assess progress of the developing fusion mass or to verify solid fusion in the absence of definitive clinical symptoms. Routine surveillance scanning should be discouraged as a means of assuaging patient anxiety about the success of the procedure and patients should be made aware of the risks and benefits if a CT scan is indicated.

We encourage spine surgeons to choose, when possible, instrumentation and implants that provide excellent visualization on MRI without artifact distortion. This is particularly important in the evaluation of adjacent level disease where MRI is the preferred imaging modality. Devices constructed of titanium offer better MRI visualization and assessment than stainless steel, but characterization of index level changes and pathology can remain blurred using conventional scanner and image acquisition algorithms [32]. Other implant materials, such as polyether ether ketone (PEEK) and bioceramics, have an established history of safe use in other areas of orthopedics and offer excellent MRI visualization [33–35]. Priority should be given to programs investigating the range of MRI compatible biomaterials for more widespread use in the spine to replace ferromagnetic instrumentation and implants.

Due to the retrospective design of our study, we were restricted to computing postoperative imaging event prevalence. Advanced imaging incidence rates could not be computed from this administrative database. However, it is our hope that this foundational work will provide the impetus for future prospective research in different spine surgical indications to estimate postoperative imaging incidence rates.

The findings of this study are also limited by the use of records from a single, geographically-specific healthcare system based on claims identified by anatomic- and imaging-specific CPT codes. It is unclear what impact this has on the generalizability of our findings as use of claims data has been found to underreport imaging utilization [36]. Additionally, since the results of the current study are restricted to CT and MRI prevalence estimates, we could not delineate the reasons why imaging was prescribed. Nonetheless, it remains essential that patient safety be given high priority when selecting the appropriate imaging modality to evaluate the postoperative spine.

## Conclusions

We detected a high frequency of CT utilization following complex spine surgery. There is emerging evidence of an increased cancer risk due to ionizing radiation exposure with CT. Thus, in the setting of complex spine surgery, actions to mitigate this risk should be considered and include reducing nonessential scans, using the lowest possible radiation dose protocols, exerting greater selectivity in monitoring the developing fusion construct, and adopting non-ferromagnetic implant biomaterials that facilitate MRI postoperatively.

## Additional file

Additional file 1: Anatomic-specific Current Procedural Terminology (CPT) codes. (DOCX 17 kb)

## Abbreviations

CAGR: compound annual growth rate
CPT: current procedural terminology
CT: computed tomography
HIPAA: health insurance portability and accountability act
MRI: magnetic resonance imaging
PEEK: polyether ether ketone
US: United States
