# Supplementary material for: Utilization of CT scanning associated with complex spine surgery
Source: BMC Musculoskelet Disord. 2017 Jan 31;18:52. doi: 10.1186/s12891-017-1420-9 (PMC5282647; doi:10.1186/s12891-017-1420-9)
Supplement: Additional file 1: — Anatomic-specific Current Procedural Terminology (CPT) codes. (DOCX 17 kb) [file 12891_2017_1420_MOESM1_ESM.docx]

**Additional file 1: Appendix**

**Cervical Spinal Fusion**

22600 Arthrodesis, posterior or posterolateral technique, single level; cervical below C2 segment

22554 Arthrodesis, anterior interbody technique, including minimal discectomy to prepare interspace (other than for decompression); cervical below C2

22551 Arthrodesis, anterior interbody, including disc space preparation, discectomy, osteophytectomy and decompression of spinal cord and/or nerve roots; cervical below C2

22552 Arthrodesis, anterior interbody, including disc space preparation, discectomy, osteophytectomy and decompression of spinal cord and/or nerve roots; cervical below C2, each additional interspace (List separately in addition to code for separate procedure)

22590 Arthrodesis, posterior technique, craniocervical (occiput-C2)

**Lumbar Spinal Fusion**

0309T Arthrodesis, pre-sacral interbody technique, including disc space preparation, discectomy, with posterior instrumentation, with image guidance, includes bone graft, when performed, lumbar, L4-L5 interspace (List separately in addition to code for primary procedure)

0334T Sacroiliac joint stabilization for arthrodesis, percutaneous or minimally invasive (indirect visualization), includes obtaining and applying autograft or allograft (structural or morselized), when performed, includes image guidance when performed (eg, CT or fluoroscopic) Code Deleted 2015-01-01

22586 Arthrodesis, pre-sacral interbody technique, including disc space preparation, discectomy, with posterior instrumentation, with image guidance, includes bone graft when performed, L5-S1 interspace

27279 Arthrodesis, sacroiliac joint, percutaneous or minimally invasive (indirect visualization), with image guidance, includes obtaining bone graft when performed, and placement of transfixing device

22630 Arthrodesis, posterior interbody technique, including laminectomy and/or discectomy to prepare interspace (other than for decompression), single interspace; lumbar

22632 Arthrodesis, posterior interbody technique, including laminectomy and/or discectomy to prepare interspace (other than for decompression), single interspace; each additional interspace (List separately in addition to code for primary procedure)

22633 Arthrodesis, combined posterior or posterolateral technique with posterior interbody technique including laminectomy and/or discectomy sufficient to prepare interspace (other than for decompression), single interspace and segment; lumbar

22634 Arthrodesis, combined posterior or posterolateral technique with posterior interbody technique including laminectomy and/or discectomy sufficient to prepare interspace (other than for decompression), single interspace and segment; each additional interspa

22612 Arthrodesis, posterior or posterolateral technique, single level; lumbar (with lateral transverse technique, when performed)

22558 Arthrodesis, anterior interbody technique, including minimal discectomy to prepare interspace (other than for decompression); lumbar

0195T Arthrodesis, pre-sacral interbody technique, disc space preparation, discectomy, without instrumentation, with image guidance, includes bone graft when performed; L5-S1 interspace

0196T Arthrodesis, pre-sacral interbody technique, disc space preparation, discectomy, without instrumentation, with image guidance, includes bone graft when performed; L4-L5 interspace (List separately in addition to code for primary procedure)

**Other Spinal Procedures**

0171T Insertion of posterior spinous process distraction device (including necessary removal of bone or ligament for insertion and imaging guidance), lumbar; single level

0172T Insertion of posterior spinous process distraction device (including necessary removal of bone or ligament for insertion and imaging guidance), lumbar; each additional level (List separately in addition to code for primary procedure)

22318 Open treatment and/or reduction of odontoid fracture(s) and or dislocation(s) (including os odontoideum), anterior approach, including placement of internal fixation; without grafting

22319 Open treatment and/or reduction of odontoid fracture(s) and or dislocation(s) (including os odontoideum), anterior approach, including placement of internal fixation; with grafting

22840 Posterior non-segmental instrumentation (eg, Harrington rod technique, pedicle fixation across 1 interspace, atlantoaxial transarticular screw fixation, sublaminar wiring at C1, facet screw fixation) (List separately in addition to code for primary procedure)

22841 Internal spinal fixation by wiring of spinous processes (List separately in addition to code for primary procedure)

22842 Posterior segmental instrumentation (eg, pedicle fixation, dual rods with multiple hooks and sublaminar wires); 3 to 6 vertebral segments (List separately in addition to code for primary procedure)

22843 Posterior segmental instrumentation (eg, pedicle fixation, dual rods with multiple hooks and sublaminar wires); 7 to 12 vertebral segments (List separately in addition to code for primary procedure)

22844 Posterior segmental instrumentation (eg, pedicle fixation, dual rods with multiple hooks and sublaminar wires); 13 or more vertebral segments (List separately in addition to code for primary procedure)

22845 Anterior instrumentation; 2 to 3 vertebral segments (List separately in addition to code for primary procedure)

22846 Anterior instrumentation; 4 to 7 vertebral segments (List separately in addition to code for primary procedure)

22847 Anterior instrumentation; 8 or more vertebral segments (List separately in addition to code for primary procedure)

22848 Pelvic fixation (attachment of caudal end of instrumentation to pelvic bony structures) other than sacrum (List separately in addition to code for primary procedure)

22849 Reinsertion of spinal fixation device

22851 Application of intervertebral biomechanical device(s) (eg, synthetic cage(s), methylmethacrylate) to vertebral defect or interspace (List separately in addition to code for primary procedure)

**Discectomy**

0274T Percutaneous laminotomy/laminectomy (interlaminar approach) for decompression of neural elements, (with or without ligamentous resection, discectomy, facetectomy and/or foraminotomy), any method, under indirect image guidance (eg, fluoroscopic, CT), with or without the use of an endoscope, single or multiple levels, unilateral or bilateral; cervical or thoracic

0275T Percutaneous laminotomy/laminectomy (interlaminar approach) for decompression of neural elements, (with or without ligamentous resection, discectomy, facetectomy and/or foraminotomy), any method, under indirect image guidance (eg, fluoroscopic, CT), with or without the use of an endoscope, single or multiple levels, unilateral or bilateral; lumbar.

22526 Percutaneous intradiscal electrothermal annuloplasty, unilateral or bilateral including fluoroscopic guidance; single level

62287 Decompression procedure, percutaneous, of nucleus pulposus of intervertebral disc, any method utilizing needle based technique to remove disc material under fluoroscopic imaging or other form of indirect visualization, with the use of an endoscope, with discography and/or epidural injection(s) at the treated level(s), when performed, single or multiple levels, lumbar.

This includes endoscopic approach. Do not report 62287 in conjunction with 62267,

62290, 62311, 77003, 77012, 72295, when performed at same level. For non-needle based technique for percutaneous decompression of nucleus pulposus of intervertebral disc, see 0274T, 0275T

62292 Injection procedure for chemonucleolysis, including discography, intervertebral disc, single or multiple levels, lumbar

63020 Laminotomy (hemilaminectomy), with decompression of nerve root(s), including partial facetectomy, foraminotomy and/or excision of herniated intervertebral disc; 1 interspace, cervical.

For bilateral procedure, report 63020 with modifier 50

63030 Laminotomy (hemilaminectomy), with decompression of nerve root(s), including partial facetectomy, foraminotomy and/or excision of herniated intervertebral disc; 1 interspace, lumbar. For bilateral procedure, report 63030 with modifier 50.

63040 Laminotomy (hemilaminectomy), with decompression of nerve root(s), including partial facetectomy, foraminotomy and/or excision of herniated intervertebral disc, reexploration, single interspace; cervical. For bilateral procedure, report 63040 with modifier 50.

63042 Laminotomy (hemilaminectomy), with decompression of nerve root(s), including partial facetectomy, foraminotomy and/or excision of herniated intervertebral disc, reexploration, single interspace; lumbar. For bilateral procedure, report 63042 with modifier 50.

63055 Transpedicular approach with decompression of spinal cord, equina and/or nerve root(s) (eg, herniated intervertebral disc), single segment; thoracic

63056 Transpedicular approach with decompression of spinal cord, equina and/or nerve root(s) (eg, herniated intervertebral disc), single segment; lumbar (including transfacet, or lateral extraforaminal approach) (eg, far lateral herniated intervertebral disc)

63075 Discectomy, anterior, with decompression of spinal cord and/or nerve root(s), including osteophytectomy; cervical, single interspace. Do not report 63075 in conjunction with 22554, even if performed by separate individuals. To report anterior cervical discectomy and interbody fusion at the same level during the same session, use 22551

63077 Discectomy, anterior, with decompression of spinal cord and/or nerve root(s), including osteophytectomy; thoracic, single interspace

**Laminectomy / Decompression**

63170 Laminectomy with myelotomy (eg, Bischof or DREZ type), cervical, thoracic, or thoracolumbar

63172 Laminectomy with drainage of intramedullary cyst/syrinx; to subarachnoid space

63173 Laminectomy with drainage of intramedullary cyst/syrinx; to peritoneal or pleural space

63180 Laminectomy and section of dentate ligaments, with or without dural graft, cervical; 1 or 2 segments

63182 Laminectomy and section of dentate ligaments, with or without dural graft, cervical; more than 2 segments

63185 Laminectomy with rhizotomy; 1 or 2 segments

63190 Laminectomy with rhizotomy; more than 2 segments

63191 Laminectomy with section of spinal accessory nerve.

63194 Laminectomy with cordotomy, with section of 1 spinothalamic tract, 1 stage; cervical

63195 Laminectomy with cordotomy, with section of 1 spinothalamic tract, 1 stage; thoracic

63196 Laminectomy with cordotomy, with section of both spinothalamic tracts, 1 stage; cervical

63197 Laminectomy with cordotomy, with section of both spinothalamic tracts, 1 stage; thoracic

63198 Laminectomy with cordotomy with section of both spinothalamic tracts, 2 stages within 14 days; cervical

63199 Laminectomy with cordotomy with section of both spinothalamic tracts, 2 stages within 14 days; thoracic

63200 Laminectomy, with release of tethered spinal cord, lumbar

63250 Laminectomy for excision or occlusion of arteriovenous malformation of spinal cord; cervical

63251 Laminectomy for excision or occlusion of arteriovenous malformation of spinal cord; thoracic

63252 Laminectomy for excision or occlusion of arteriovenous malformation of spinal cord; thoracolumbar

63265 Laminectomy for excision or evacuation of intraspinal lesion other than neoplasm, extradural; cervical

63266 Laminectomy for excision or evacuation of intraspinal lesion other than neoplasm, extradural; thoracic

63267 Laminectomy for excision or evacuation of intraspinal lesion other than neoplasm, extradural; lumbar

63268 Laminectomy for excision or evacuation of intraspinal lesion other than neoplasm, extradural; sacral

63270 Laminectomy for excision of intraspinal lesion other than neoplasm, intradural; cervical

63271 Laminectomy for excision of intraspinal lesion other than neoplasm, intradural; thoracic

63272 Laminectomy for excision of intraspinal lesion other than neoplasm, intradural; lumbar

63273 Laminectomy for excision of intraspinal lesion other than neoplasm, intradural; sacral

63709 Repair of dural/cerebrospinal fluid leak or pseudomeningocele, with laminectomy

63740 Creation of shunt, lumbar, subarachnoid-peritoneal, -pleural, or other; including laminectomy

63001 Laminectomy with exploration and/or decompression of spinal cord and/or cauda equina, without facetectomy, foraminotomy or discectomy (eg, spinal stenosis), 1 or 2 vertebral segments; cervical

63003 Laminectomy with exploration and/or decompression of spinal cord and/or cauda equina, without facetectomy, foraminotomy or discectomy (eg, spinal stenosis), 1 or 2 vertebral segments; thoracic

63005 Laminectomy with exploration and/or decompression of spinal cord and/or cauda equina, without facetectomy, foraminotomy or discectomy (eg, spinal stenosis), 1 or 2 vertebral segments; lumbar, except for spondylolisthesis

63011 Laminectomy with exploration and/or decompression of spinal cord and/or cauda equina, without facetectomy, foraminotomy or discectomy (eg, spinal stenosis), 1 or 2 vertebral segments; sacral

63012 Laminectomy with removal of abnormal facets and/or pars inter-articularis with decompression of cauda equina and nerve roots for spondylolisthesis, lumbar (Gill type procedure)

63015 Laminectomy with exploration and/or decompression of spinal cord and/or cauda equina, without facetectomy, foraminotomy or discectomy (eg, spinal stenosis), more than 2 vertebral segments; cervical

63016 Laminectomy with exploration and/or decompression of spinal cord and/or cauda equina, without facetectomy, foraminotomy or discectomy (eg, spinal stenosis), more than 2 vertebral segments; thoracic

63017 Laminectomy with exploration and/or decompression of spinal cord and/or cauda equina, without facetectomy, foraminotomy or discectomy (eg, spinal stenosis), more than 2 vertebral segments; lumbar

63045 Laminectomy, facetectomy and foraminotomy (unilateral or bilateral with decompression of spinal cord, cauda equina and/or nerve root[s], [eg, spinal or lateral recess stenosis]), single vertebral segment; cervical

63046 Laminectomy, facetectomy and foraminotomy (unilateral or bilateral with decompression of spinal cord, cauda equina and/or nerve root[s], [eg, spinal or lateral recess stenosis]), single vertebral segment; thoracic

63047 Laminectomy, facetectomy and foraminotomy (unilateral or bilateral with decompression of spinal cord, cauda equina and/or nerve root[s], [eg, spinal or lateral recess stenosis]), single vertebral segment; lumbar

**Computed Tomography**

72125 Computed tomography, cervical spine; without contrast material

72126 Computed tomography, cervical spine; with contrast material

72127 Computed tomography, cervical spine; without contrast material, followed by contrast material(s) and further sections

72128 Computed tomography, thoracic spine; without contrast material

72129 Computed tomography, thoracic spine; with contrast material

72130 Computed tomography, thoracic spine; without contrast material, followed by contrast material(s) and further sections

72131 Computed tomography, lumbar spine; without contrast material

72132 Computed tomography, lumbar spine; with contrast material

72133 Computed tomography, lumbar spine; without contrast material, followed by contrast material(s) and further sections

**Magnetic Resonance**

72141 Magnetic Resonance Imaging of neck spine w/o dye

72142 Magnetic Resonance Imaging of neck spine w/dye

72146 Magnetic Resonance Imaging of chest spine w/o dye

72147 Magnetic Resonance Imaging of chest spine w/dye

72148 Magnetic Resonance Imaging of lumbar spine w/o dye

72149 Magnetic Resonance Imaging of lumbar spine w/dye

72156 Magnetic Resonance Imaging of neck spine w/o & w/dye

72158 Magnetic Resonance Imaging of lumbar spine w/o & w/dye

72195 Magnetic Resonance Imaging of pelvis w/o dye

72196 Magnetic Resonance Imaging of pelvis w/dye

72197 Magnetic Resonance Imaging of pelvis w/o & w/dye
